# Veganism: an extended theory of planned behavior framework incorporating ethical, environmental, and sociodemographic determinants

**DOI:** 10.3389/fnut.2026.1761348

**Published:** 2026-02-04

**Authors:** Ece Öneş, Cansu Gençalp, Gizem Avcı, Simge Sipahi, Meryem Kahrıman, Salim Yılmaz, Murat Baş

**Affiliations:** 1 Department of Nutrition and Dietetics, Faculty of Health Sciences, Acibadem Mehmet Ali Aydinlar University, Istanbul, Türkiye; 2 Department of Nutrition and Dietetics, Graduate School of Health Sciences, Acibadem Mehmet Ali Aydinlar University, Istanbul, Türkiye; 3 Department of Healthcare Management, Faculty of Health Sciences, Acibadem Mehmet Ali Aydinlar University, Istanbul, Türkiye

**Keywords:** anti-speciesism, ethical concerns, health belief, theory of planned behavior, vegan diet, veganism

## Abstract

**Background/objectives:**

Despite increasing global interest in veganism, integrative models that incorporate ethical, environmental, and psychosocial determinants within the Theory of Planned Behavior (TPB) remain limited in Türkiye. This study aimed to extend the TPB by including ethical, environmental, and health-related motivations to better explain individuals’ intentions and behaviors related to adopting and maintaining a vegan diet.

**Methods:**

A cross-sectional online survey was conducted among adults in Türkiye who identified with or engaged in veganism. Twelve latent variables were assessed using validated scales, and the extended model was tested through structural equation modeling with additional robustness procedures, including spline adjustments, PCA, Elastic Net regularization, and instrumental variable analyses.

**Results:**

Subjective norms and perceived behavioral control significantly predicted vegan intention, with subjective norms emerging as the strongest determinant. Ethical motivation strongly predicted intention but did not directly predict actual adherence. Unexpectedly, environmental and health motivations were negatively associated with adherence. Women reported stronger intentions despite perceiving lower social support.

**Conclusion:**

This study broadens the TPB by integrating ethical, normative, and psychosocial dimensions that explain vegan intentions beyond traditional predictors. Findings underscore the importance of moral identity, perceived social expectations, and contextual factors in shaping sustainable dietary behaviors.

## 1 Introduction

Climate change, characterized by extreme weather events and rising sea levels, is primarily driven by anthropogenic greenhouse gas emissions. Food production systems are among the largest contributors to greenhouse gas emissions. In addition to greenhouse gas emissions, they also exert significant impacts through land use deforestation, and the desiccation of wetlands (1). This growing environmental crisis has drawn global attention to the urgent need for sustainable food systems and dietary transitions. This situation highlighted the need to think about sustainable food production system and nutrition, and the Food and Agriculture Organization (FAO) and the World Health Organization (WHO) defined the concept of sustainable diets. Their key emphasis was on the fact that plant-based diets have lower environmental impacts, while animal-based diets have higher greenhouse gas emissions (2). In addition, the EAT-Lancet Commission supported these findings when introducing the Planetary Health Diet, which has a low environmental impact and is based mostly on plant-based foods (1). In light of these global developments, veganism has gained increasing recognition as one of the most prominent sustainable dietary patterns (3).

Veganism is not just a dietary pattern, but a comprehensive way of life that aims to exclude all forms of exploitation and cruelty to animals for food, clothing, or any other purpose. It involves avoiding all animal-derived foods such as meat (including fish, red meat, and poultry), dairy products, eggs, and honey, while embracing plant-based alternatives instead (4). This lifestyle is increasingly popular globally. A study conducted by YouGov and Veganuary in 2025 indicated that approximately 25.8 million people worldwide had tried veganism (5). Moreover, in 2021, searches for ‘vegan food near me’ on Google increased by more than 5,000% (6). According to a 2020 study by Sia Insight, the combined proportion of vegetarians and vegans in Turkey is estimated to be below 5%, with approximately 80,000 individuals identifying specifically as vegan (7). While these figures illustrate the global and national rise in veganism, they also highlight the importance of understanding the psychological, social, and ethical mechanisms that drive individuals to adopt and maintain this lifestyle.

Previous research has indicated that the main reasons for adopting a vegan diet are centered on animal welfare, protecting the planet by minimizing environmental impacts, and health reasons (8–10). One of the main goals of veganism is to end animal exploitation and mitigate the environmental destruction that results from it (3). Similarly, in a qualitative study conducted with Generation Z in Türkiye, animal rights, sustainability, environment, healthy lifestyle and impact of influencers were determined to be important factors (11). In another qualitative study conducted in Türkiye, ethical, cognitive/social, health and ecological factors were identified as important motivations (12). Additionally, one of the biggest reasons for choosing a vegan diet is its perceived association with a reduced risk of chronic diseases like diabetes, heart disease, and obesity. However, the long-term consequences of eliminating animal products from the diet, such as vitamin B12, iron, calcium, vitamin D, iodine, zinc, and omega-3 fatty acids, are also significant obstacles (13). Sociodemographic components such as sex, marital status, education and income status are also important predictors in choosing a vegan diet (14, 15). These diverse motivations—ethical, environmental, health-related, and social—can be conceptually integrated within a behavioral framework that explains how such factors shape individuals’ intentions and actions.

The Theory of Planned Behavior (TPB) (16) provides a valuable lens for understanding such complex human behaviors. According to TPB, behavioral intention is determined by three key constructs: attitudes toward the behavior, subjective norms, and perceived behavioral control. Together, these components explain the likelihood of performing a specific behavior. When applied to veganism, this framework allows for the examination of how ethical beliefs, environmental awareness, and health motivations form attitudes toward a vegan lifestyle; how social norms and stigma influence decision-making; and how perceived barriers affect behavioral control.

Despite the growing literature on vegan motivations (8–10), quantitative studies that integrate ethical and sociodemographic factors into TPB remain scarce, particularly in Türkiye. Previous research (11) has primarily focused on qualitative explorations, leaving a gap in understanding how psychological, ethical, and social constructs interact to predict vegan dietary behaviors. Therefore, this study aimed to extend the TPB by integrating ethical, environmental, and health-related factors to explain individuals’ intentions and behaviors toward adopting and maintaining a vegan diet.

## 2 Literature review and hypotheses

**Figure 1:**
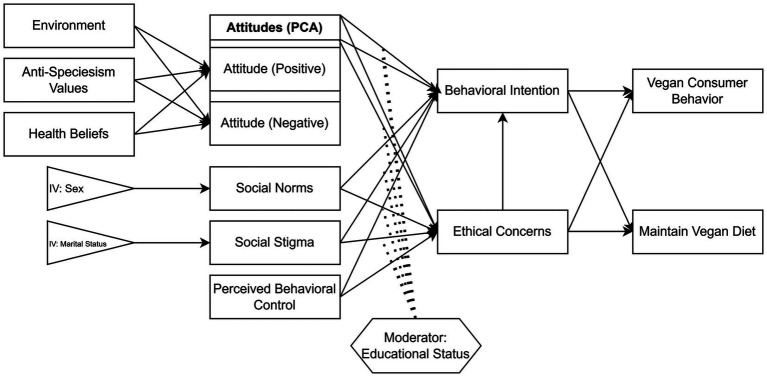
Conceptual model of psychosocial, normative, and ethical determinants of vegan lifestyle adoption and maintenance.

Grounded in the interdisciplinary nature of vegan dietary behavior, this study integrates multiple theoretical perspectives to capture the moral, psychological, and social dimensions influencing individuals’ adoption and maintenance of a vegan lifestyle. To explore the determinants that shape individuals’ adoption and maintenance of a vegan lifestyle, an integrative conceptual model (Figure 1) was developed by integrating core value-based, psychosocial, normative, and ethical constructs derived from well-established behavioral frameworks. This model draws upon multiple theoretical foundations, including the Theory of Planned Behavior (TPB) (16), the Value-Belief-Norm Theory (17), the Animal Rights Theory (18), the Inherent Value Theory (19), the Health Belief Model (20), and the Stigmatization Theory (21). By integrating these theoretical perspectives, the model offers a comprehensive framework that links ethical reasoning, perceived social expectations, and individual control beliefs to behavioral intentions related to vegan lifestyle choices.

### 2.1 Attitude, subjective norms, and perceived behavioral control

Ajzen’s (16) Theory of Planned Behavior (TPB) provides a comprehensive framework for explaining how individuals develop intentions that lead to specific behaviors. According to the TPB, individuals’ intentions are shaped by three core constructs: attitude, which reflects an individual’s overall positive or negative evaluation of a behavior; subjective norms, which reflect the perceived social expectations or pressures to perform it; and perceived behavioral control, which reflects an individual’s perceived capability or control over performing and maintaining the behavior (16). The theory posits that the more favorable an individual’s attitudes, the stronger their perceived control over performing the behavior, and the more supportive the social norms within their environment, the greater their intention to engage in that behavior (22). In a study conducted among vegan consumers, attitude, perceived behavioral control, and social norms were all found to have significant positive effects on purchase intention (23).

In addition to perceived behavioral control and social norms, ethical concern has been consistently identified as a central motivational factor in the adoption of vegan and plant-based diets, particularly through its influence on attitudes toward animal welfare, environmental sustainability, and social responsibility (23). Previous research demonstrates that individuals with stronger ethical concerns tend to evaluate veganism more favorably, perceive greater moral obligation to avoid animal products, and report stronger intentions to adopt or maintain vegan diets (23, 24). Building upon these theoretical foundations and in the context of vegan dietary behavior, the following hypotheses were developed:

*H1*: Stronger positive attitudes predict higher behavioral intention.

*H2*: Greater perceived behavioral control predicts higher behavioral intention.

*H3*: Stronger social norms predict higher behavioral intention.

*H4*: Higher behavioral intention predicts greater engagement in vegan consumer behaviors.

*H5*: Higher behavioral intention predicts greater adherence to a vegan diet.

*H6*: Higher ethical concerns predict greater engagement in vegan consumer behaviors

### 2.2 Environmental concerns

The influence of environmental concerns on vegan attitudes can be explained within the framework of Stern et al.’s (17) Value-Belief-Norm Theory and its ecological worldview. As individuals become more environmentally aware, their understanding of the environmental costs associated with animal-based food production—such as greenhouse gas emissions, land use, and water consumption—also deepens. This enhanced awareness and moral concern for environmental sustainability may ultimately foster more favorable attitudes toward adopting a vegan lifestyle (17). Based on this theoretical framework, the following hypotheses were developed.

*H7*: Higher environmental concerns are associated with more positive attitudes toward veganism.

*H8*: Higher environmental concerns are associated with stronger adherence intentions.

### 2.3 Anti-speciesism values

The influence of anti-speciesism values on attitudes is theoretically grounded in Singer’s animal rights philosophy (18) and Regan’s inherent value theory (19). These perspectives assert that all sentient beings possess moral worth and that ethical consideration should not be limited by species boundaries (18, 19). Individuals who strongly endorse anti-speciesist values are therefore more likely to make lifestyle choices that prioritize animal welfare, thereby fostering more positive attitudes toward veganism (3). Based on this theoretical framework, the following hypotheses were developed.

*H9*: Stronger anti-speciesism values are associated with more positive attitudes toward veganism.

*H10*: Stronger anti-speciesism values are associated with stronger adherence intentions.

### 2.4 Health beliefs

The influence of health motivations on vegan attitudes can be conceptualized within the framework of the Health Belief Model (HBM). The HBM, originally developed to explain and predict health-related behaviors, proposes that such behaviors are determined by individuals’ perceptions of susceptibility, severity, benefits, barriers, cues to action, and self-efficacy (20). Accordingly, beliefs regarding the potential health benefits of a vegan diet—such as a reduced risk of cardiovascular diseases, cancer prevention, and longevity—may positively shape individuals’ attitudes toward adopting a vegan lifestyle (25). In line with this theoretical framework, the following hypotheses were formulated.

*H11*: Stronger health beliefs are associated with more positive attitudes toward veganism.

*H12*: Stronger health beliefs are associated with stronger adherence intentions.

### 2.5 Stigma

Goffman’s stigmatization theory explains how certain individuals or groups in society become negatively labeled, marginalized, and devalued because of their distinct characteristics, identities, or behaviors (21). In this context, experiences of social exclusion, ridicule, or criticism faced by vegan individuals may negatively affect both their behavioral intentions and ethical motivations (26). Based on this theoretical framework, the following hypotheses were developed.

*H13*: Higher stigma predicts higher behavioral intention and stronger ethical concerns.

### 2.6 Sociodemographic factors

In addition to the main theoretical constructs, sociodemographic variables were incorporated into the model to provide a more comprehensive understanding of individual differences. The theoretical justification for choosing sex as an instrumental variable for social norms lies in the notion that gender roles systematically shape perceptions of normative expectations. According to Eagly and Wood’s Social Role Theory (27), women tend to experience stronger social pressures to engage in communal or relational behaviors, whereas men are typically oriented toward agentic roles. Such role differentiation may lead to sex-based variations in the social perception of vegan behavior.

The rationale for including marital status as an instrumental variable for social stigma is that the institution of marriage substantially broadens individuals’ social networks and role expectations. Married individuals typically maintain more complex and interdependent social interaction networks through their spouses, children, and extended family circles (28). Consequently, individuals who adopt unconventional lifestyles—such as veganism—may face heightened risks of social pressure, prejudice, or stigmatization from their social environment. Even the anticipation of adopting veganism may evoke concerns about disapproval or criticism from close social ties (26).

Finally, a higher educational level is associated with enhanced information-processing capacity, critical thinking skills, and the ability to adopt multiple perspectives. This may lead to differences in how individuals interpret and respond to social influences, attitudes, and behavioral intentions. Therefore, educational level was designated as a moderating variable in the relationships examined (29). Based on these theoretical considerations, the following hypotheses were developed regarding the roles of sex, marital status, and educational level in shaping vegan attitudes and behaviors.

*H14*: Female sex predicts higher perceived social norm pressure regarding veganism.

*H15*: Being married predicts higher perceived stigma regarding veganism.

*H16*: Education level moderates the relationships between attitudes, social norms, and perceived behavioral control with ethical concerns.

H17: There are significant indirect effects of sex on behavioral intention, intention to adopt vegan consumer behaviors, and adherence to a vegan diet.

*H18*: There are significant indirect effects of marital status on behavioral intention, intention to adopt consumer behaviors, and adherence to a vegan diet.

## 3 Materials and methods

This study employed a cross-sectional survey design to examine psychosocial, ethical, and environmental determinants of vegan dietary intentions and behaviors. Ethical approval for the involvement of human subjects in this study was granted by the Acibadem Mehmet Ali Aydinlar University Medical Research Ethics Committee (ATADEK 2024-8/326, 16.05.2024). Individuals who identified with or were engaged in veganism to varying degrees were included in the study. Prior to the study, participants were asked to provide informed consent electronically. Participation was voluntary, and participants could withdraw at any time without providing a reason. The study was conducted in accordance with the principles of the Declaration of Helsinki.

Twelve latent variables were derived from the observed variables listed in Table 1, and the measurement items were adapted from validated instruments used in previous studies (3, 23). All items were measured on a five-point Likert scale, ranging from 1 (strongly disagree) to 5 (strongly agree).

**Table 1:** Latent variables and items.

Latent variables	Items
SN	SN1: My vegan friends/family think that I should maintain a strict vegan diet
	SN2: My colleagues think that I should maintain a strict vegan diet
	SN3: My close friends think that I should maintain a strict vegan diet
	SN4: My spouse/partner thinks that I should maintain a strict vegan diet
	SN5: My family thinks that I should maintain a strict vegan diet
AT	AT1: Maintaining a strict vegan diet is valuable to me
	AT2: Maintaining a strict vegan diet is good for me
	AT3: Maintaining a strict vegan diet is a pleasant behavior for me
	AT4: Maintaining a strict vegan diet is enjoyable for me
BI	BI1: I am ready to control my strict vegan diet even when vegan food is not available.
	BI2: I intend to maintain a strict vegan diet.
	BI3: I want to maintain a strict vegan diet.
	BI4: I hope to maintain a strict vegan diet.
MVD	MVD1: It is easy to eat a nutritionally balanced diet while maintaining a strict vegan diet.
	MVD2: Maintaining a strict vegan diet minimizes my risk of developing long-term health complications.
	MVD3: Maintaining a strict vegan diet encourages me to eat a healthier diet.
	MVD4: I have more energy while maintaining a strict vegan diet.
	MVD5: I feel physically better while maintaining a strict vegan diet.
EC	EC1: I try not to contribute to the profit of companies and brands that cause animal suffering because it is wrong.
	EC2: I believe that consuming or using animal products is wrong because animals are not our property.
	EC3: I have adopted a vegan lifestyle for ethical reasons.
PBC	PBC1: By using my knowledge of ingredients and label reading, I can determine whether a product is safe.
	PBC2: Vegan products are clearly labeled.
	PBC3: I am confident in my ability to identify vegan foods safely and to ask necessary questions about contamination.
	PBC4: Vegan products are easily accessible
	PBC5: I am prepared and organized regarding my diet
ASV	ASV1: I try not to contribute to the profit of companies and brands that cause animal suffering because it is wrong.
	ASV2: I believe that consuming or using animal products is wrong because animals are not our property.
	ASV3: I feel upset when I see wild animals in cages in zoos.
	ASV4: It is wrong to kill animals to make clothing.
	ASV5: The use of animals in rodeos and circuses is cruel.
	ASV6: I have seriously considered becoming vegan to save animals’ lives.
	ASV7: It is unacceptable to breed any animal for human consumption.
HB	HB1: I do not need meat or other animal products to be healthy.
	HB2: Following a plant-based diet and avoiding animal products is better for my health.
	HB3: I have thought that I would be much healthier if I gave up animal products.
	HB4: I have adopted a vegan lifestyle for health reasons.
ENV	ENV1: I have adopted a vegan lifestyle for environmental reasons.
	ENV2: Avoiding meat and animal products reduces my carbon footprint and helps protect the environment.
	ENV3: A vegan lifestyle greatly helps reduce water, air, and soil pollution, thereby preserving and saving the environment for future generations.
ATA	ATA1: Maintaining a strict vegan diet is impossible for me.
	ATA2: Maintaining a strict vegan diet is unenjoyable for me.
	ATA3: Maintaining a strict vegan diet is bad for me.
	ATA4: Maintaining a strict vegan diet is harmful for me.
	ATA5: Maintaining a strict vegan diet is unpleasant for me.
STIG	STIG1: (Because I am vegan) I have been unable to attend meals due to the lack of suitable food.
	STIG2: (Because I am vegan) I have been called a bad person.
	STIG3: (Because I am vegan) I have been labeled.
	STIG4: (Because I am vegan) I have frequently faced harassment.
	STIG5: I have been singled out for being vegan.
	STIG6: (Because I am vegan) I have been mocked or laughed at.
	STIG7: (Because I am vegan) I have been frequently teased or ridiculed.
VCB	VCB1: I purchase vegan foods.
	VCB2: I choose to buy products from brands that avoid animal testing.
	VCB3: I always purchase cruelty-free products.
	VCB4: When I need to buy clothes, I choose not to buy any items made from animal skin, fur, or other animal-derived materials.
	VCB5: When I need to buy furniture, I choose not to buy any items made from animals.

SN, social norms; AT, attitude; BI, behavioral intention; MVD, maintain vegan diet; EC, ethical concerns; PBC, perceived behavioral control; ASV, anti-speciesism values; HB, health beliefs; ENV, environmental concerns; ATA, attitude toward adherence to a vegan diet; STIG, stigma; VCB, vegan consumer behavior.

All statistical analyses were performed using R version 4.4.2 (see Supplementary Text S1 for detailed package information). Exploratory Factor Analysis (EFA) and Confirmatory Factor Analysis (CFA) were conducted for all latent constructs. Skewness and kurtosis diagnostics indicated significant departures from normality for all latent variables (see Supplementary Table S1 for detailed statistics). Consequently, non-parametric Spearman correlations and bias-corrected and accelerated (BCa) bootstrap confidence intervals were employed to ensure robust inference. Discriminant validity was examined using the Fornell–Larcker criterion. The linearity assumption of the structural equation model was assessed using Ramsey RESET tests, and where necessary, natural spline transformations were applied to improve model fit and address nonlinear relationships. To manage potential multicollinearity, Principal Component Analysis (PCA) was applied to highly correlated variables, and Elastic Net regularization was employed to further minimize residual collinearity in mediation and interaction models. For instrumental variable (IV) validity, three standard assumptions—relevance, exclusion restriction, and exogeneity—were systematically tested to confirm the appropriateness of gender and marital status as instruments for Social Norms and Stigma, respectively. In the customized PROCESS framework, bias-corrected and accelerated (BCa) confidence intervals were estimated using 20,000 bootstrap samples. All model adjustments (spline, PCA, and Elastic Net corrections) were integrated into the resampling procedure. Statistical significance was determined when the 95% confidence interval did not include zero, and R^2^ values for the structural components were reported to assess model performance.

## 4 Results

In total, 447 individuals participated in this study. A detailed overview of the sample covering demographic characteristics, perceptions related to environmental and health considerations, and vegan status is given in Table 2. Most participants were women (78.08%). A significant number of participants believed that their monthly shopping was harming the environment to a moderate (30.87%) to high degree (36.24%). More than half (51.29%) valued product healthiness in high importance and almost half (41.83%) indicated that they paid close attention to how environmentally friendly the items they bought were. Among the participants, 49.22% indicated that at least one person in their close social circle was vegan. With respect to vegan status, 48.32% of the participants identified as vegan, whereas a smaller segment reported being non-vegan but inclined toward vegan products (14.32%).

**Table 2:** Sociodemographic characteristics of participants.

Characteristics	Items	*n*	%
Sex	Female	349	78.08
	Male	98	21.92
Perceived environmental impact of monthly shopping	None/very little	16	3.58
	Low	53	11.86
	Moderate	138	30.87
	High	162	36.24
	Very high	78	17.45
Attention to the eco-friendliness of purchased products	None/very little	21	4.70
	Low	48	10.74
	Moderate	119	26.62
	High	187	41.83
	Very high	72	16.11
Attention to the healthiness of purchased products	None/very little	4	0.89
	Low	15	3.36
	Moderate	102	22.82
	High	232	51.90
	Very high	94	21.03
Education level	High school	60	13.42
	Associate degree	33	7.38
	Bachelor’s degree	219	48.99
	MSc. and Ph.D.	135	30.2
Marital status	Single	258	57.72
	Married	189	42.28
Occupation	Unemployed	39	8.72
	Homemaker	18	4.03
	Retired	15	3.36
	Self-employed	55	12.30
	Student	60	13.42
	Private sector employee	175	39.15
	Public sector employee	65	14.54
	Large-scale employer	20	4.47
Income	Below the minimum wage	39	8.72
	Around the minimum wage	44	9.84
	Student and pocket money	41	9.17
	TL 20.000–30.000	76	17
	TL 30.000–40.000	74	16.55
	TL 13.400 TL-20.000	70	15.66
	TL ≥ 40.000 TL	103	23.04
Household size	1	77	17.23
	2	170	38.03
	3	110	24.61
	4	61	13.65
	5 or more	29	6.49
Living situation	Single, living with child/children	4	0.89
	Living with spouse/partner, no children	124	27.74
	Living with spouse/partner, with children	80	17.90
	Living alone	77	17.23
	Single, living with family members	129	28.86
	Living with roommates	33	7.38
Presence of vegans in close social circle	No	196	43.85
	Yes	220	49.22
	Not sure	31	6.94
Vegan status	Yes	216	48.32
	No	167	37.36
	Not vegan, but interested in vegan products	64	14.32

For each latent construct, Table 3 displays the factor loadings derived from exploratory and confirmatory factor analyses, as well as the eigenvalues (SS), Cronbach’s alpha values, average variance extracted (AVE), composite reliability (CR), total variance explained (TVE), and eigenvalues (SS). Parallel analysis was used to assess the factor structure of the scales that made up the theoretical framework of the study. For Attitudes, Behavioral Intention, Maintain Vegan Diet, Ethical Concerns, Attitude Toward Adherence to a Vegan Diet, and Environment, the analyses first proposed a one-factor structure; for Social Norms, Anti-Speciesism Values, Health Beliefs, Stigma, and Intention, a two-factor structure; and for Perceived Behavioral Control, a three-factor structure. Nonetheless, a one-factor solution was retained for all subscales after considering item content, theoretical explanations, factor loadings more than 0.50 on a single factor in exploratory factor analysis and explained variance ratios greater than 50% for all latent constructs. No items were eliminated since none of them showed low loadings. The KMO values for Environment (0.61) and Ethical Concerns (0.69) were marginal but deemed acceptable given adequate variance explanation and high factor loadings. All constructs showed significant results from the Bartlett’s test (*p* < 0.05). Additionally, a common method bias analysis using all the items of the scale revealed no significant concern since the single-factor model explained only 49% of total variance.

**Table 3:** Construct validity and reliability of latent variables.

Latent variable	Item	Exploratory factor analysis results			Confirmatory factor analysis and reliability results			
		EFA Loading	SS Loading	TVE (%)	CFA Loading	CR	AVE	Alpha
SN	SN1	0.71	3.38	68	0.695	0.909	0.670	0.904
	SN2	0.88			0.914			
	SN3	0.91			0.934			
	SN4	0.77			0.723			
	SN5	0.82			0.797			
AT	AT1	0.94	3.73	93	0.945	0.983	0.934	0.982
	AT2	0.98			0.982			
	AT3	0.99			0.984			
	AT4	0.95			0.955			
BI	BI1	0.93	3.67	92	0.937	0.979	0.922	0.978
	BI2	0.99			0.988			
	BI3	0.97			0.968			
	BI4	0.94			0.948			
MVD	MVD1	0.86	4.18	84	0.877	0.963	0.838	0.962
	MVD2	0.93			0.918			
	MVD3	0.89			0.887			
	MVD4	0.94			0.943			
	MVD5	0.95			0.951			
EC	EC1	0.84	2.42	81	0.826	0.924	0.803	0.920
	EC2	0.99			0.945			
	EC3	0.85			0.913			
PBC	PBC1	0.84	2.56	51	0.828	0.831	0.508	0.826
	PBC2	0.53			0.513			
	PBC3	0.91			0.875			
	PBC4	0.56			0.500			
	PBC5	0.67			0.758			
ASV	ASV1	0.84	4.68	67	0.805	0.919	0.627	0.927
	ASV2	0.84			0.939			
	ASV3	0.80			0.634			
	ASV4	0.80			0.615			
	ASV5	0.78			0.595			
	ASV6	0.84			0.931			
	ASV7	0.82			0.928			
HB	HB1	0.90	2.85	71	0.950	0.900	0.702	0.898
	HB2	0.93			0.949			
	HB3	0.91			0.854			
	HB4	0.58			0.526			
ENV	ENV1	0.50	2.10	70	0.512	0.868	0.701	0.830
	ENV2	0.97			0.951			
	ENV3	0.95			0.969			
ATA	ATA1	0.92	4.32	86	0.914	0.969	0.864	0.969
	ATA2	0.90			0.894			
	ATA3	0.97			0.970			
	ATA4	0.91			0.925			
	ATA5	0.94			0.944			
STIG	STIG1	0.60	4.73	68	0.580	0.933	0.669	0.931
	STIG2	0.79			0.765			
	STIG3	0.88			0.853			
	STIG4	0.89			0.880			
	STIG5	0.74			0.718			
	STIG6	0.91			0.936			
	STIG7	0.90			0.932			
VCB	VCB1	0.69	3.19	64	0.808	0.896	0.634	0.891
	VCB2	0.87			0.878			
	VCB3	0.89			0.869			
	VCB4	0.77			0.704			
	VCB5	0.76			0.704			

SN, social norms; AT, attitude; BI, behavioral intention; MVD, maintain vegan diet; EC, ethical concerns; PBC, perceived behavioral control; ASV, anti-speciesism values; HB, health beliefs; ENV, environmental concerns; ATA, attitude toward adherence to a vegan diet; STIG, stigma; VCB, vegan consumer behavior. Common bias SS loading, 27.81 and proportional variance, 0.49.

All subscales had strong and stable CFA loadings, ranging from 0.51 to 0.99, according to the factor loadings, composite reliability, and Cronbach’s alpha values derived from the confirmatory factor analysis. Cronbach’s alpha values were also within the range of 0.83 to 0.98, as were composite reliability values. Because the items accurately measured the intended latent structure, each latent construct was considered to have high construct validity and internal consistency. The inclusion of these dimensions in the measurement model is supported by the fact that the majority of factor loadings exceeded 0.70 and that both CR and alpha values were above accepted psychometric standards. The Fornell–Larcker criterion was used to evaluate discriminant validity prior to testing the measurement model. The diagonal values of each construct, representing the square root of its AVE, were higher than the correlations of that construct with every other latent variable in the matrix. For example, the AVE for Social Norms had a square root of 0.818, higher than its associations with Attitudes (0.366), Behavioral Intention (0.356), and Maintain Vegan Diet (0.412). Likewise, the values were 0.936 for Attitudes, 0.960 for Behavioral Intention, 0.916 for Maintain Vegan Diet, 0.896 for Ethical Concerns, 0.713 for Perceived Behavioral Control, 0.792 for Anti-Speciesism Values, 0.838 for Environment, 0.837 for Health Beliefs, 0.930 for Attitude Toward Adherence to a Vegan Diet, 0.818 for Stigma, and 0.796 for Vegan Consumer Behavior. Sufficient discriminant validity was demonstrated across all dimensions as the square root of the AVE for each construct was higher than its correlations with other constructs in the same row or column. As a result, the theoretically suggested multidimensional structure of the measurement model was empirically supported by the findings that each latent construct was statistically unique from the others and that there was no substantial overlap across variables.

The normality assumption of the latent variables was not met. The primary criterion for the normality test was skewness and kurtosis statistics, which were represented as *z*-scores in relation to their standard errors. Both skewness and kurtosis values must be within the ±1.96 range for a distribution to be considered normal. Skewness and kurtosis values were determined to be outside of these statistical bounds with a sample size of *n* = 417. For instance, the Anti-Speciesism Values construct had skewness = −1.56 and kurtosis = 4.95, but the Intention construct had skewness = −1.20 and kurtosis = 3.84. Consequently, none of the constructs met the normal distribution assumption. Furthermore, some of the most notable values were Z-skewness = 9.43 and Z-kurtosis = 17.87 for Social Norms and Z-skewness = −3.86 and Z-kurtosis = 7.15 for Attitudes. These results precluded the use of parametric methods, leading to the adoption of robust estimation techniques in the analysis that followed.

**Figure 2:**
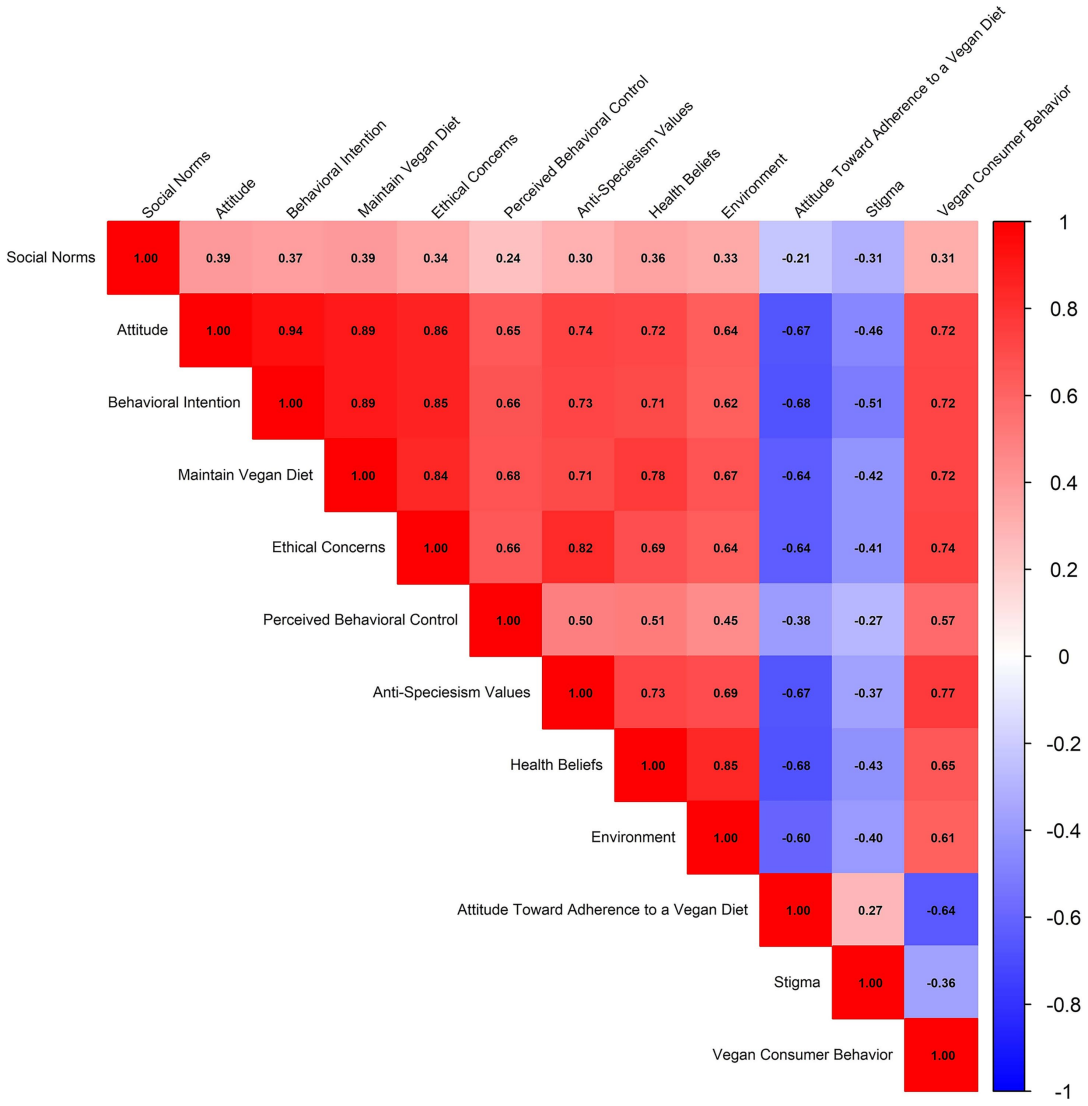
Correlations among latent variables.

Spearman’s rank correlation was used in Figure 2 to identify the relationships among variables.

Nearly all the constructs of the model showed moderate-to-high and statistically significant positive correlations, according to the results of the Spearman correlation analysis, which examined the relationships between latent variables. Strong associations were observed between Anti-Speciesism Values and Ethical Concerns (*r* = 0.82), Attitude and Ethical Concerns (*r* = 0.86), Behavioral Intention and Intention (*r* = 0.72), Behavioral Intention and Maintain Vegan Diet (*r* = 0.89), and Attitude and Behavioral Intention (*r* = 0.94). Negative correlations were observed between Stigma and Attitude (*r* = −0.46), and between Attitude Toward Adherence to a Vegan Diet and Attitude (*r* = −0.67). The hypothesized causal paths in the structural model are empirically supported by the substantial intercorrelations among the latent variables.

The impact of basic demographic variables on the model constructs was examined to assess the validity of instrumental variables. Regression analysis was used to determine the relationships of gender and marital status with the potentially dependent latent variables. The findings showed that only Social Norms, Anti-Speciesism Values, and Behavioral Intention were substantially correlated with gender (*p* < 0.05), with no significant correlations detected with the other latent constructs. Only Social Norms and Stigma were substantially correlated with marital status; no significant correlations were found for the other dependent constructs. Furthermore, gender and marital status were considered exogenous (*p* > 0.05) as they did not directly influence the subsequent endogenous factors. The model only included gender as an instrumental variable for Social Norms and marital status as an instrumental variable for Stigma considering these findings and the theoretical framework. The reasoning behind this is that an instrumental variable should not relate to other endogenous/dependent constructs in the model but should only substantially predict the associated latent construct. An instrument’s validity may be weakened and exogeneity compromised if it is linked to several constructs. Since each latent construct had only one suitable instrumental variable and there were no surplus instruments, the overidentification test—a diagnostic used when excessive instruments over constrain the model—was not performed. As a result, there was no problem with overidentification, and the test was considered unnecessary. Furthermore, additional demographic variables were not considered for instrumental variable selection since they were significantly related with all latent constructs in the model (*p* < 0.05), such as the presence of vegans in the close social circle. Conversely, education level was coded from 1 to 4 (low to high) and included as a moderator variable in theoretically relevant paths, with analysis conducted accordingly.

Ramsey RESET tests revealed significant linearity violations in the initial models. Natural spline transformations (df = 5) were applied to address these violations, successfully resolving nonlinearity in the Attitude and Maintain Vegan Diet models. For models with residual nonlinearity, BCa bootstrap inference was employed, which remains robust under modest model misspecification. Diagnostic plots are presented in Supplementary Figure S1, with detailed test statistics in Supplementary Table S2.

Variance inflation factor (VIF) analysis identified multicollinearity concerns in interaction models. A three-stage correction strategy was implemented: (1) principal component analysis for attitude-related predictors, (2) Elastic Net regularization for outcome models, reducing maximum VIF from 2068 to 4.8, and (3) integration of all corrections into the bootstrap resampling procedure. Detailed VIF statistics are provided in Supplementary Table S3.

The instrumental variable analysis provides crucial insights into the causal foundations of the model. The significant negative coefficient for sex (*β* = −0.470, 95% CI [−0.706, −0.253]) indicates that females report substantially lower perceptions of social norms supporting vegan dietary behaviors compared to males. However, when total and indirect effects are considered, the results reveal the opposite pattern for actual intention and behavior: female gender is associated with significantly higher vegan intention (*β* = 0.194, 95% CI [0.119, 0.304]) and a greater likelihood of maintaining a vegan diet (*β* = 0.224, 95% CI [0.121, 0.357]) compared to males. This finding suggests that, despite perceiving less social support, women are more likely than men to intend to adopt and sustain vegan dietary behaviors, potentially reflecting stronger individual motivations or alternative social influences outside mainstream norms. The marital status effect (*β* = 0.258, 95% CI [0.050, 0.457]) demonstrates that married individuals experience significantly higher levels of vegan-related stigma than single individuals, possibly reflecting increased social scrutiny from extended family networks or partner influence dynamics (Table 4).

**Table 4:** Model path coefficients estimated with natural spline, PCA, and elastic net correction for linearity and multicollinearity violations.

Path	Coef.	95% CI lower	95% CI upper
Instrumental variables			
Sex (female) → Social norms	−0.470*	−0.706	−0.253
Marital status (married) → Stigma	0.258*	0.050	0.457
Attitude formation paths			
Environment → Attitude	0.384	−0.501	1.400
Anti-speciesism values → Attitude	0.602	−0.435	1.658
Health beliefs → Attitude	0.007	−0.942	0.877
Attitude toward adherence to a vegan diet paths			
Environment → Attitude toward adherence to a vegan diet	−1.052*	−1.832	−0.262
Anti-speciesism values → Attitude toward adherence to a vegan diet	−0.154	−0.991	0.866
Health Beliefs → Attitude Toward Adherence to a Vegan Diet	−0.711*	−1.487	−0.048
Mediator-to-outcome paths			
PC1_attitude → Behavioral intention	−0.708	−0.829	0.728
PC2_attitude → Behavioral intention	−0.382	−0.771	0.415
Perceived behavioral control → Behavioral intention	0.524*	0.404	0.651
Social norms → Behavioral Intention	0.840*	0.449	1.869
Stigma → Behavioral intention	0.297	−0.275	2.293
Education level → Behavioral intention	−0.004	−0.077	0.069
PC1_attitude → Ethical concerns	−0.578	−0.710	0.581
PC2_attitude → Ethical concerns	−0.566	−1.117	0.450
Perceived behavioral control → Ethical concerns	0.566*	0.437	0.692
Social norms → Ethical concerns	0.266	−0.049	0.813
Stigma → Ethical concerns	−0.502	−2.367	0.059
Education level → Ethical concerns	−0.013	−0.075	0.053
Elastic net final outcome paths			
Behavioral intention → Vegan consumer behavior	−0.439*	−1.281	−0.026
Ethical concerns → Vegan consumer behavior	1.135*	0.667	2.028
Behavioral intention → Maintain vegan diet	0.760*	0.510	0.906
Ethical concerns → Maintain vegan diet	0.139	0.000	0.411
Indirect effects			
Sex → Social norms → Behavioral intention → Intention	0.108*	0.020	0.403
Sex → Social norms → Behavioral intention → Maintain vegan diet	0.187*	0.098	0.340
Sex → Social norms → Ethical Concerns → Intention	0.302*	0.187	0.658
Sex → Social norms → Ethical Concerns → Maintain vegan diet	0.037	−0.000	0.127
Marital Status → Stigma → Behavioral Intention → Intention	−0.095*	−0.573	−0.006
Marital status → Stigma → Behavioral intention → Maintain vegan diet	−0.165*	−0.453	−0.055
Marital status → Stigma → Ethical concerns → Intention	−0.078*	−0.456	−0.009
Marital status → Stigma → Ethical concerns → Maintain vegan diet	−0.010	−0.083	0.000
Total effects			
Total: Sex → Vegan consumer behavior	0.194*	0.119	0.304
Total: Sex → Maintain vegan diet	0.224*	0.121	0.357
Total: Marital status → Vegan consumer behavior	−0.017	−0.275	0.062
Total: Marital status → Maintain vegan diet	−0.175*	−0.457	−0.057

Sex and marital status are coded as dummy variables (Female = 1, Male = 0; Married = 1, Single = 0), and education level is modeled as a continuous ordinal variable (1 = Secondary education, 2 = High school, 3 = Undergraduate, 4 = Graduate degree). Bootstrap confidence intervals were computed using the bias-corrected and accelerated (BCa) method with 20,000 replications. Statistical significance (marked by *) indicates a 95% confidence interval that does not include zero. Inal model estimates incorporate natural spline transformations (df = 5) to address nonlinear relationships, as well as principal component analysis (PCA) and elastic net regularization to control for multicollinearity (maximum VIF re-duced from 2068 to 4.8). Spline-corrected and regularized models yielded substantial increases in explained variance for attitude (*R*^2^: 0.59 → 0.65), attitude toward adherence to a vegan diet (0.48 → 0.67), behavioral intention (0.69 → 0.81), ethical concerns (0.78 → 0.84), and maintain vegan diet (0.75 → 0.80). All reported coefficients and indirect effects reflect these final, optimized models.

The attitude formation results reveal a striking paradox in how different motivational factors influence general attitudes versus specific adherence attitudes. For general vegan attitudes, none of the traditional predictors (environment, anti-speciesism, health) achieved statistical significance, with coefficients ranging from near-zero (health: *β* = 0.007) to moderate but non-significant effects (anti-speciesism: *β* = 0.602, environment: *β* = 0.384). This suggests that general attitudes toward veganism may be influenced by factors not captured in this model or may represent a more complex, multi-dimensional construct. In stark contrast, attitudes toward vegan diet adherence show significant negative effects from both environmental concerns (*β* = −1.052, 95% CI [−1.832, −0.262]) and health beliefs (*β* = −0.711, 95% CI [−1.487, −0.048]). This counterintuitive finding suggests that individuals with stronger environmental and health motivations may paradoxically develop more negative attitudes toward strict adherence to vegan diets. This could reflect realistic appraisals of the practical challenges involved in maintaining a vegan diet, where individuals who are more knowledgeable about environmental and health issues also better understand the difficulties of perfect adherence (Table 4).

The mediation analysis reveals that perceived behavioral control (*β* = 0.524, 95% CI [0.404, 0.651]) and social norms (*β* = 0.840, 95% CI [0.449, 1.869]) are the primary drivers of behavioral intention to maintain a vegan diet. The social norms effect is particularly robust, representing the strongest single predictor in the model. Notably, the principal components derived from attitude measures (PC1: *β* = −0.708; PC2: *β* = −0.382) show negative but non-significant effects, suggesting that after controlling for behavioral control and social factors, attitudes may have limited direct influence on behavioral intentions. The absence of significant effects from stigma (*β* = 0.297, CI includes zero) and education (*β* = −0.004, CI includes zero) indicates that these factors do not meaningfully predict behavioral intention after accounting for other variables. This is particularly noteworthy for education, suggesting that knowledge or cognitive sophistication alone does not enhance vegan dietary intentions (Table 4).

For ethical concerns, perceived behavioral control again emerges as the strongest predictor (*β* = 0.566, 95% CI [0.437, 0.692]), with a magnitude nearly identical to its effect on behavioral intention. This consistency suggests that feelings of control over vegan behaviors are fundamental to both practical intentions and ethical reasoning. Social norms show a weaker, non-significant effect on ethical concerns (*β* = 0.266, CI includes zero), indicating that ethical reasoning may be less susceptible to social influence than behavioral intentions. The negative coefficient for stigma (*β* = −0.502, CI [−2.367, 0.059]) approaches significance and suggests that experiencing vegan-related stigma may reduce ethical concerns about non-vegan behaviors, possibly through psychological defensive mechanisms or cognitive dissonance reduction (Table 4).

The regularized final outcome models reveal perhaps the most theoretically significant finding: a strong negative relationship between behavioral intention and vegan consumer behavior (*β* = −0.439, 95% CI [−1.281, −0.026]). This paradoxical result suggests that individuals with stronger intentions to maintain a vegan diet may simultaneously report lower general intentions toward vegan-related behaviors. This could reflect a trade-off where dietary focus comes at the expense of broader vegan lifestyle commitments, or alternatively, may indicate that diet-specific intentions represent a more realistic, constrained form of veganism that acknowledges practical limitations. Conversely, ethical concerns show a strong positive relationship with vegan consumer behavior (*β* = 1.135, 95% CI [0.667, 2.028]), indicating that ethical reasoning supports broader vegan commitments beyond diet alone. For vegan diet maintenance, behavioral intention shows the expected strong positive effect (*β* = 0.760, 95% CI [0.510, 0.906]), while ethical concerns show a weaker, non-significant effect (*β* = 0.139, CI [0.000, 0.411]), suggesting that practical intentions matter more than ethical reasoning for sustained dietary behavior (Table 4).

The indirect effects reveal complex gender-based pathways in vegan behavior adoption. Through the social norms pathway, female sex shows multiple significant mediation effects: positive effects on both vegan consumer behavior (*β* = 0.108, 95% CI [0.020, 0.403]) and diet maintenance (*β* = 0.187, 95% CI [0.098, 0.340]) via behavioral intention, as well as a positive effect on vegan consumer behavior via ethical concerns (*β* = 0.302, 95% CI [0.187, 0.658]). These findings indicate that, although women perceive less social support for vegan behaviors, their overall likelihood of intending and maintaining a vegan diet is higher than men, possibly due to alternative motivational or normative sources. For marital status, the total effects show a non-significant impact on vegan consumer behavior (*β* = −0.017, CI includes zero) but a significant negative effect on diet maintenance (*β* = −0.175, 95% CI [−0.457, −0.057]), suggesting that being married may create barriers to sustaining a vegan diet, possibly due to increased stigma or conflicting family expectations (Table 4).

The successful application of natural splines, PCA, and elastic net regularization demonstrates the importance of addressing methodological violations in complex behavioral models. The substantial *R*-squared improvements (ranging from 0.59 → 0.65 to 0.75 → 0.80) indicate that these corrections not only resolved statistical problems but also enhanced the model’s explanatory power. The reduction of maximum VIF from 2068 to 4.8 represents a dramatic improvement in multicollinearity control, enhancing the reliability and interpretability of all reported coefficients. These findings collectively paint a picture of vegan behavior adoption as a complex, multi-pathway process where traditional assumptions about motivation, gender, and social influence require substantial revision. The paradoxical relationships observed—particularly the negative association between behavioral intention and general vegan intention—suggest that vegan behavior research must account for trade-offs and competing priorities within individuals’ value systems and practical constraints (Table 4).

The model performance statistics demonstrate excellent explanatory power across all structural equation components (Table 5). The attitude formation models achieved substantial explained variance, with the general attitude model accounting for 65.2% of variance (95% CI [56.8, 70.6%]) and the attitude toward adherence model explaining 67.1% of variance (95% CI [57.6, 72.7%]). The mediation models showed even stronger performance, with the behavioral intention model explaining 74.0% of variance (95% CI [68.4, 77.3%]) and the ethical concerns model achieving 74.9% explained variance (95% CI [69.8, 78.8%]). The final outcome model for vegan diet maintenance demonstrated the highest explanatory power at 79.7% (95% CI [74.6, 83.2%]), indicating robust predictive validity for sustained vegan dietary behavior. These R-squared values substantially exceed typical benchmarks for behavioral research, with all models surpassing the 0.65 threshold considered indicative of strong explanatory power. The narrow confidence intervals across all models suggest stable and reliable parameter estimates, supporting the robustness of the natural spline, PCA, and elastic net corrections implemented to address methodological violations. The progressive increase in explained variance from attitude formation (*R*^2^ ≈ 0.65) through mediation processes (*R*^2^ ≈ 0.74) to final outcomes (*R*^2^ ≈ 0.80) indicates that the theoretical model successfully captures the complexity of vegan dietary decision-making processes.

**Table 5:** Model performance: *R*-squared values with bootstrap confidence intervals.

Models	*R* ^2^	95% CI lower	95% CI upper
Attitude	0.652	0.568	0.706
Attitude toward adherence to a vegan diet	0.671	0.576	0.727
Behavioral intention	0.740	0.684	0.773
Ethical concerns	0.749	0.698	0.788
Maintain vegan diet	0.797	0.746	0.832

## 5 Discussion

The findings of this study validate and expand the Theory of Planned Behavior (TPB) model for understanding vegan dietary choices. The study supports Ajzen’s model (16) by showing that both subjective norms and perceived behavioral control (PBC) affect people’s decisions to follow a vegan diet. The study results show that participants in this research group respond most strongly to social expectations from their environment because subjective norms proved to be the leading predictor (*β* = 0.840) compared to PBC (*β* = 0.524). Consistent with D’Souza et al. (23), our findings suggest that social norms play an important role in adopting plant-based diets, particularly in cultures where shared values and group belonging are important.

Perceived behavioral control, as expected, also played a substantial role in shaping intention. This is consistent with prior research indicating that individuals who feel confident in their ability to access, prepare, and adhere to vegan diets are more likely to intend and follow through on such behaviors (30, 31). This idea is especially important in dietary settings where limited access to vegan foods or cultural barriers can make healthy eating harder to achieve (32).

In our study, ethical motivation stood out as a strong predictor of vegan consumer behavior (*β* = 1.135), highlighting how deeply values shape people’s commitment to their diets. This finding is consistent with earlier research showing that individuals driven by ethical concerns—especially those related to animal welfare and anti-speciesist beliefs—are more likely to develop a stronger vegan identity (3, 33). Interestingly, our study revealed a notable divergence: while ethical motivation strongly shaped individuals’ intentions, it did not directly predict actual vegan behavior. This challenges the common assumption that ethical reasoning automatically leads to consistent action and instead points to a clear value–action gap (34, 35). In other words, even deeply held ethical values may not always translate into behavior due to emotional, social, or structural barriers—underscoring the need to consider mediating factors such as behavioral control or habit in future research.

Perhaps the most unexpected finding was the negative relationship between behavioral intention and vegan consumer behavior (*β* = −0.439). This runs counter to the Theory of Planned Behavior (TPB), which proposes that intention is the most direct predictor of behavior (16). One explanation may be that participants expressed strong intentions to follow a vegan diet for short-term or specific reasons—such as improving their health—without embracing veganism as a broader lifestyle. Previous research has made similar distinctions between “health vegans” and “ethical vegans” (9), with the latter group showing more stable, identity-based behaviors (36). This pattern also points to a limitation of the TPB in explaining value-driven lifestyles, where behavior may depend more on identity, habits, and emotions than on intention alone (37).

Contrary to common assumptions, our study found that environmental and health motivations were negatively linked to dietary adherence (*β* = −1.052 and *β* = −0.711, respectively). Although these factors are often cited as the initial reasons people choose plant-based diets (38), our results suggest they may not sustain long-term commitment to veganism. One possible explanation is that these motivations are outcome-focused and conditional; for instance, if the expected health or environmental benefits are not experienced, individuals may be more likely to abandon the behavior (39). Health-based messages can also trigger psychological resistance, particularly when they clash with cultural food norms or the enjoyment of eating (33, 40). Overall, these findings suggest that ethical motivations—grounded in stable moral values—may provide a stronger and more enduring foundation for dietary commitment than utilitarian motivations (41, 42).

Our gender-related findings offer a more nuanced view of how men and women differ in their motivations and behaviors toward veganism. Although women in our study perceived less social support for veganism, they reported a stronger intention to maintain a vegan diet (*β* = 0.224). This unexpected pattern suggests that women may rely more on internal values and moral reasoning than on external social approval. Ruby (43) observed a similar trend, noting that empathy and care-based reasoning often play a key role in women’s decisions to reduce or avoid meat consumption. In contrast, men may be less open to plant-based eating because of identity-related norms that link meat consumption with masculinity (44, 45). These insights suggest that plant-based diet campaigns could be more effective if they explicitly address gender norms and identity-related barriers.

Although stigma was not a statistically significant predictor in our study, previous research and theory suggest it may still play an important role in shaping vegan-related behaviors and identity. Studies have shown that vegans often experience stigma and social exclusion, which can discourage them from adopting or maintaining a vegan identity (3). The pressure to conform to mainstream eating habits may be especially strong in cultures where meat is closely linked to tradition and social rituals. Moreover, even though it was not directly examined here, experiences such as witnessing animal cruelty or facing a personal health crisis have been found to spark lasting dietary change (23). Future research should further explore how these experiences influence the transition to veganism.

Taken together, our model showed strong explanatory power (*R*^2^ > 0.65), supporting the usefulness of the Theory of Planned Behavior (TPB) in predicting vegan dietary intentions. However, the findings also point to some limitations of the traditional TPB framework in capturing value-based and identity-driven behaviors. As highlighted by Klöckner (31) and Sniehotta et al. (37), expanding the model to include factors such as moral identity, emotional engagement, habit strength, and situational barriers could provide a more comprehensive understanding of behavior. Moreover, the interplay between ethical, health, and environmental motivations deserves further exploration, as different motives may drive different forms of veganism and varying levels of behavioral consistency (42).

Although this study provides valuable insights, several limitations should be noted. First, its cross-sectional design prevents drawing causal conclusions about the relationship between motivations and behavior, making it unclear whether these motivations sustain long-term vegan commitment (16). Second, the use of self-reported measures may have introduced social desirability bias, especially in responses related to ethical beliefs (39). Third, because participants were primarily recruited online, the sample may overrepresent younger and more educated individuals who are already interested in veganism. This could limit the generalizability of the findings. Future research should adopt longitudinal designs, include more diverse and representative samples, and examine additional psychological factors to better capture the complexity of vegan behavior.

## 6 Conclusion

These findings reinforce the relevance of the Theory of Planned Behavior in explaining vegan dietary intentions and underscore the significant influence of social and ethical factors on food-related decisions. Subjective norms emerged as the strongest predictor of behavioral intention, while ethical concerns—rather than health or environmental motivations—showed the most robust association with actual vegan consumer behavior. The unexpected negative relationship between behavioral intention and vegan consumer behavior suggests that intention-based models may be insufficient for capturing value-driven dietary choices. By integrating subjective norms, perceived behavioral control, and moral values, the study enhances understanding of value-driven dietary choices. The integration of ethical, normative, and psychosocial constructs provides an extended behavioral framework that captures the multidimensional nature of vegan lifestyle adoption.

From a practical standpoint, these findings suggest that public health campaigns promoting plant-based diets should prioritize ethical messaging and social norm cultivation over health or environmental appeals alone. Community-based interventions that foster supportive social environments may be particularly effective given the strong influence of subjective norms. Additionally, addressing gender-specific barriers—particularly the finding that women maintain stronger intentions despite perceiving lower social support—could enhance the effectiveness of dietary behavior change programs.

Future studies should adopt longitudinal designs to examine how motivations and intentions translate into sustained behavioral change over time. Cross-cultural comparisons would clarify whether the observed patterns—particularly the paradoxical negative relationship between behavioral intention and vegan consumer behavior—generalize beyond the Turkish context. Incorporating objective behavioral measures, qualitative methods, and experimental designs would further strengthen causal inference and provide deeper insight into the psychological mechanisms underlying vegan lifestyle adoption.

## Data Availability

The raw data supporting the conclusions of this article will be made available by the authors, without undue reservation.
