# Supplementary material for: Veganism: an extended theory of planned behavior framework incorporating ethical, environmental, and sociodemographic determinants
Source: Front Nutr. 2026 Feb 4;13:1761348. doi: 10.3389/fnut.2026.1761348 (PMC12915333; doi:10.3389/fnut.2026.1761348)
Supplement: Supplementary file 1 [file Data_Sheet_1.docx]

**Supplementary Text S1: Statistical Software and R Packages**

All statistical analyses were conducted using R version 4.4.2 (R Core Team, 2024). The following packages were utilized:

**Data Management and Manipulation:** readxl (v1.4.3) (1) for Excel file import, dplyr (v1.1.4) (2) for data transformation.

**Statistical Analysis:** boot (v1.3-30) (3) for bootstrap resampling with BCa confidence intervals (20,000 iterations), car (v3.1-2) (4) for VIF calculations and diagnostic tests, lmtest (v0.9-40) (5)for RESET linearity tests, splines (base R) for natural spline transformations.

**Psychometric Analysis:** lavaan (v0.6-17) (6) for confirmatory factor analysis, semTools (v0.5-6) (7) for measurement invariance testing, psych (v2.4.3) (8) for reliability analysis and descriptive statistics.

**Visualization:** ggplot2 (v3.5.1) (9) for diagnostic plots, corrplot (v0.92) (10) for correlation matrices, gridExtra (v2.3) (11) for multi-panel figures.

**Normality Assessment:** moments (v0.14.1) (12) for skewness and kurtosis calculations, Hmisc (v5.1-3) (13) for correlation significance testing.

**Supplementary Text S2: Methodological Notes and Justifications**

**S2.1 Normality Violations and Robust Estimation**

All 12 latent variables exhibited significant departures from univariate normality based on Z-score transformations of skewness and kurtosis (Table S1).

Supplementary Table S1. Normality Assessment Results

| **Variable** | **Skewness** | **Kurtosis** | **Z-Skewness** | **Z-Kurtosis** | **Normal (S)** | **Normal (K)** |
| --- | --- | --- | --- | --- | --- | --- |
| Social Norms | 1.09 | 4.12 | 9.43 | 17.87 | No | No |
| Attitude | -0.45 | 1.65 | -3.86 | 7.15 | No | No |
| Behavioral Intention | -0.29 | 1.48 | -2.50 | 6.44 | No | No |
| Maintain Vegan Diet | -0.48 | 1.82 | -4.15 | 7.88 | No | No |
| Ethical Concerns | -0.73 | 2.13 | -6.29 | 9.23 | No | No |
| Perceived Behavioral Control | -0.68 | 2.63 | -5.88 | 11.41 | No | No |
| Anti-Speciesism Values | -1.56 | 4.95 | -13.48 | 21.48 | No | No |
| Health Beliefs | -0.60 | 2.23 | -5.16 | 9.68 | No | No |
| Environment | -0.71 | 2.66 | -6.16 | 11.56 | No | No |
| Attitude Toward Adherence | 0.83 | 2.40 | 7.20 | 10.41 | No | No |
| Stigma | -0.43 | 2.20 | -3.76 | 9.55 | No | No |
| Vegan Consumer Behavior | -1.21 | 3.84 | -10.44 | 16.68 | No | No |

*N = 447. Normal = Yes if |Z| < 1.96 (p > .05). All variables exhibited significant departures from normality, justifying the use of Spearman correlations and BCa bootstrap confidence intervals.*

Anti-Speciesism Values showed the most severe deviation (Z-skewness = -13.48, Z-kurtosis = 21.48), indicating strong negative skew with heavy tails. These violations preclude reliance on parametric assumptions for standard errors and confidence intervals. To address these violations, we employed: (1) Spearman rank-order correlations instead of Pearson correlations for bivariate relationships, ensuring robustness to non-normality and outliers; (2) Bias-corrected and accelerated (BCa) bootstrap confidence intervals with 20,000 resamples for all indirect effects, which do not assume normality of the sampling distribution and provide more accurate coverage under non-normal conditions (14).

**S2.2 Linearity Diagnostics and Remediation**

Ramsey RESET tests revealed significant linearity violations in all five key regression models (Table S2).

Supplementary Table S2. Linearity Diagnostic Results (Ramsey RESET Test)

| **Model** | **F-statistic** | **p-value** | **NS p-value** | **Resolved** |
| --- | --- | --- | --- | --- |
| Attitude Model | 7.84 | <.001 | .151 | Yes |
| Behavioral Intention Model | 36.05 | <.001 | <.001 | No* |
| Ethical Concerns Model | 42.44 | <.001 | <.001 | No* |
| Vegan Consumer Behavior Model | 5.00 | .007 | <.001 | No* |
| Maintain Vegan Diet Model | 21.67 | <.001 | .445 | Yes |

*RESET test with powers 2-3 on fitted values. NS = Natural Splines (df = 5). *BCa bootstrap confidence intervals (20,000 iterations) used to ensure robust inference despite residual non-linearity. Visual inspection of component-plus-residual plots (Figure S1) confirmed that deviations were minor and did not substantively affect parameter estimates.*

We implemented natural spline transformations (df = 5) to address non-linearity, which successfully resolved violations in the Attitude Model (p = .151) and Maintain Vegan Diet Model (p = .445). For models with persistent non-linearity (Behavioral Intention, Ethical Concerns, Vegan Consumer Behavior), we relied on BCa bootstrap inference, which remains valid under model misspecification.

The following comprehensive visualization presents linearity assessment results for the bootstrap model examining vegan diet adoption behaviors at Figure 3. This multi-panel display includes residual plots, Q-Q plots for normality assessment, and component-plus-residual plots comparing linear (red) versus LOESS (orange) fits across all key relationships in the theoretical model. The residual plots assess homoscedasticity and linearity assumptions by examining the scatter of residuals around zero, while Q-Q plots evaluate normality of residuals. Component-plus-residual plots provide visual comparison between linear and non-parametric fits to detect potential non-linear relationships. Together, these diagnostic plots enable comprehensive evaluation of model assumptions underlying the structural equation pathways from environmental concerns, anti-speciesism values, and health beliefs through attitudes, social norms, stigma, perceived behavioral control, and ethical concerns to behavioral intentions and vegan diet maintenance outcomes (Figure S1).

Visual inspection of component-plus-residual plots (Figure S1, Panel C) confirmed that the deviations from linearity were relatively minor and concentrated at distribution extremes. The LOESS smoothing lines closely approximated linear fits across the central range of predictor values, suggesting that non-linearity does not substantively bias parameter estimates for the majority of observations.





Supplementary Figure S1. Linearity Assessment Plots for Model

**S2.3 Multicollinearity and Essential Collinearity**

VIF analysis revealed two distinct patterns (Table S3). First, main effect models showed acceptable collinearity (VIF < 5 for Attitude Model predictors; VIF approximately 1.0 for instrumental variables). Second, interaction models exhibited extremely high VIF values (up to 2068 for education-centered terms), representing essential multicollinearity inherent to interaction specifications rather than problematic redundancy.

Supplementary Table S3. Multicollinearity Assessment

| **Model** | **Variable** | **VIF** |
| --- | --- | --- |
| Attitude Model | ENVIRON_mean | 4.73 |
|  | ANTI_mean | 2.51 |
|  | HEALTH_mean | 4.85 |
| Behavioral Intention (Main Effects) | AT_pred | 26.27 |
|  | ATTITO_pred | 25.25 |
|  | PBC_mean | 1.51 |
|  | SN_pred (IV) | 1.06 |
|  | STIGMA_pred (IV) | 1.03 |
| Behavioral Intention (Interactions) | egitim_centered | 2068.39 |
|  | egitim_centered:STIGMA_pred | 961.00 |
|  | AT_pred:egitim_centered | 248.97 |
| Vegan Consumer Behavior Model | BEHINT_pred | 15.31 |
|  | ETHIC_pred | 15.31 |
| Maintain Vegan Diet Model | BEHINT_pred | 15.31 |
|  | ETHIC_pred | 15.31 |

VIF < 5: no concern; VIF 5-10: moderate; VIF > 10: severe multicollinearity. High VIF in interaction models represents essential multicollinearity (mathematical artifact of interaction specification), not problematic redundancy. IV = Instrumental Variable. Highlighted rows show instrumental variables with minimal collinearity (VIF < 1.1), indicating clean identification.

Multicollinearity assessment identified critical collinearity issues in several key model components, with variance inflation factors (VIF) reaching as high as 2068 in certain interaction models. To systematically mitigate this problem, we adopted a three-stage solution. First, for attitude-related predictors (Attitude and Attitude Toward Adherence to Vegan Diet), we applied principal component analysis, extracting two orthogonal components (PC1 and PC2) that accounted for 82.04% and 17.96% of the total variance, respectively. This step dramatically reduced VIFs from 26.5 to a range of 1.0–1.5 in the mediation models, without loss of theoretical meaning. In the final outcome models, high correlation between the two primary mediators (r = 0.86) led to VIFs of 15.3, indicating persistent multicollinearity risk. To address this, we implemented Elastic Net regularization—a hybrid of Ridge and Lasso regression—using cross-validated alpha parameters (α = 0.7 for Intention, α = 0.9 for Maintain Vegan Diet). This approach further reduced all VIFs to below 5, and enabled automatic variable selection: ethical concerns were excluded from the vegan diet maintenance model, while both mediators were retained for the intention outcome. Through this multi-level correction strategy, the highest VIF observed across all models was reduced from 2068 to 4.8, ensuring robust estimation, theoretical interpretability, and full compatibility with the Hayes-style bootstrap framework. This process allowed for accurate reporting of direct, indirect, and total effects without distortion from collinearity artifacts.

**References**

1. Wickham H, Bryan J. readxl: Read Excel Files. (2025)

2. Wickham H, Francois R, Henry L, Muller K, Vaughan D. dplyr: A Grammar of Data Manipulation. (2023)

3. Canty A, Ripley B. boot: Bootstrap R (S-Plus) Functions. R package  version 1.3-31. (2024)

4. Fox J, Weisberg S. car: Companion to Applied Regression (Version 3.1-3) [R package]. Comprehensive R Archive Network (CRAN). https://CRAN.R-project.org/package=car (Accessed March 31, 2025). (2024)

5. Zeileis A, Hothorn T. lmtest: Testing Linear Regression Models (Version 0.9-40) [R package]. Comprehensive R Archive Network (CRAN). https://CRAN.R-project.org/package=lmtest (Accessed March 31, 2025). (2024)

6. Rosseel Y. lavaan: An R Package for Structural Equation Modeling. *J Stat Softw* (2012) 48:1–36. doi: 10.18637/jss.v048.i02

7. Jorgensen TD, Pornprasertmanit S, Schoemann AM, Rosseel Y. semTools: Useful tools for structural equation modeling. (2022)

8. Revelle W. psych: Procedures for Psychological, Psychometric, and Personality Research. (2025)

9. Wickham H. “Programming with ggplot2.,” *Elegant Graphics for Data Analysis*. (2016). p. 241–253 doi: 10.1007/978-3-319-24277-4_12

10. Wei T, Simko V. R package “corrplot”: Visualization of a Correlation  Matrix (Version 0.95). (2024)

11. Auguie B. gridExtra: Miscellaneous Functions for “Grid” Graphics. (2017)

12. Lukasz Komsta, Frederick Novomestky. moments: Moments, Cumulants, Skewness, Kurtosis and Related Tests. (2022)

13. Frank E. Hmisc: Harrell Miscellaneous. (2025)

14. Efron B, Tibshirani RJ. *An Introduction to the Bootstrap*. Chapman and Hall/CRC. (1994). doi: 10.1201/9780429246593

15. Angrist JD, Pischke J-S. *Mostly Harmless Econometrics*. Princeton University Press. (2009). doi: 10.1515/9781400829828

16. Stock JH, Yogo M. “Testing for Weak Instruments in Linear IV Regression.,” *Identification and Inference for Econometric Models*. Cambridge University Press (2005). p. 80–108 doi: 10.1017/CBO9780511614491.006

17. Stok FM, de Vet E, de Ridder DTD, de Wit JBF. The potential of peer social norms to shape food intake in adolescents and young adults: a systematic review of effects and moderators. *Health Psychol Rev* (2016) 10:326–340. doi: 10.1080/17437199.2016.1155161

18. Rosenfeld DL. The psychology of vegetarianism: Recent advances and future directions. *Appetite* (2018) 131:125–138. doi: 10.1016/j.appet.2018.09.011
